# Medical Maxims

**Published:** 1874-10

**Authors:** 


Medical Maxims.—All physicians should be familiar with the following maxims of Chomel, which have become classical: The proper aim of the physician is, first, not to do harm; and second, to try to do good. The physician is not to treat diseases, but patients affected with diseases. The former of these maxims inculcates, in other words, the protection and promotion of tolerance as a fundamental rule in the practice of medicine. The latter maxim inculcates this rule with still greater emphasis.

"I was dogmatic at twenty, an observer at thirty, an empiric at forty, and now, at fifty, I no longer have any system." So said Bordeau; and he is quite right; sooner or later in science, as in life, we ought to arrive at that wisdom which sees facts as they are, and not through the glasses of preconceptions.

